# Delta Changes in [^18^F]FDG PET/CT Parameters Can Prognosticate Clinical Outcomes in Recurrent NSCLC Patients Who Have Undergone Reirradiation–Chemoimmunotherapy

**DOI:** 10.3390/biomedicines13081866

**Published:** 2025-07-31

**Authors:** Brane Grambozov, Nazanin Zamani-Siahkali, Markus Stana, Mohsen Beheshti, Elvis Ruznic, Zarina Iskakova, Josef Karner, Barbara Zellinger, Sabine Gerum, Falk Roeder, Christian Pirich, Franz Zehentmayr

**Affiliations:** 1 Department of Radiation Oncology, Paracelsus Medical University, SALK, Müllner Hauptstraße 48, A-5020 Salzburg, Austria; m.stana@salk.at (M.S.); e.ruznic@salk.at (E.R.); z.iskakova@salk.at (Z.I.); j.karner@salk.at (J.K.); s.gerum@salk.at (S.G.); f.roeder@salk.at (F.R.); f.zehentmayr@salk.at (F.Z.); 2 Division of Molecular Imaging and Theranostics, Department of Nuclear Medicine, Paracelsus Medical University, SALK, A-5020 Salzburg, Austria; nazaninzamanisiahkali@gmail.com (N.Z.-S.); m.beheshti@salk.at (M.B.); 3 Research Center for Nuclear Medicine, Tehran University of Medical Sciences, Tehran 14117-13135, Iran; 4 Institute of Pathology, Paracelsus Medical University, SALK, A-5020 Salzburg, Austria; b.zellinger@salk.at; 5 RadART—Institute for Research and Development on Advanced Radiation Technologies, Paracelsus Medical University, A-5020 Salzburg, Austria; 6 Department of Nuclear Medicine, Paracelsus Medical University, SALK, A-5020 Salzburg, Austria; c.pirich@salk.at

**Keywords:** reirradiation, non-small cell lung cancer, image biomarkers, 18F-FDG-PET-CT

## Abstract

**Background and Purpose**: Stratification based on specific image biomarkers applicable in clinical settings could help optimize treatment outcomes for recurrent non-small cell lung cancer patients. For this purpose, we aimed to determine the clinical impact of positive delta changes (any difference above zero > 0) between baseline [^18^F]FDG PET/CT metrics before the first treatment course and reirradiation. **Material/Methods:** Forty-seven patients who underwent thoracic reirradiation with curative intent at our institute between 2013 and 2021 met the inclusion criteria. All patients had histologically verified NSCLC, ECOG (Eastern Cooperative Oncology Group) ≤ 2, and underwent [^18^F]FDG PET/CT for initial staging and re-staging before primary radiotherapy and reirradiation, respectively. The time interval between radiation treatments was at least nine months. Quantitative metabolic volume and intensity parameters were measured before first irradiation and before reirradiation, and the difference above zero (>0; delta change) between them was statistically correlated to locoregional control (LRC), progression-free survival (PFS), and overall survival (OS). **Results:** Patients were followed for a median time of 33 months after reirradiation. The median OS was 21.8 months (95%-CI: 16.3–27.3), the median PFS was 12 months (95%-CI: 6.7–17.3), and the median LRC was 13 months (95%-CI: 9.0–17.0). Multivariate analysis revealed that the delta changes in SULpeak, SUVmax, and SULmax of the lymph nodes significantly impacted OS (SULpeak *p* = 0.017; SUVmax *p* = 0.006; SULmax *p* = 0.006), PFS (SULpeak *p* = 0.010; SUVmax *p* = 0.009; SULmax *p* = 0.009), and LRC (SULpeak *p* < 0.001; SUVmax *p* = 0.003; SULmax *p* = 0.003). **Conclusions:** Delta changes in SULpeak, SUVmax, and SULmax of the metastatic lymph nodes significantly impacted all clinical endpoints (OS, PFS and LRC) in recurrent NSCLC patients treated with reirradiation. Hence, these imaging biomarkers could be helpful with regard to patient selection in this challenging clinical situation.

## 1. Introduction

Lung cancer remains the leading cause of cancer-related death, affecting approximately 1.8 million people worldwide [1]. Despite advances in treatment, 20–44% [2,3] of patients still experience locoregional recurrence [2,4,5,6]. This makes thoracic reirradiation, especially in combination with chemoimmunotherapy, an increasingly important treatment modality not only in the palliative but also in the curative setting, as it can potentially prolong survival in selected patients [2,3,4,5,6,7].

Nevertheless, as previously discussed in a systematic review [2] thoracic reirradiation remains a challenge for radiation oncologists, particularly due to the difficulty of balancing efficacy and minimizing toxicity. Hence, patient stratification is necessary and can play a crucial role in optimizing treatment outcomes. Recent studies have highlighted the importance of positron emission tomography/computed tomography (PET/CT) using 2-deoxy-2-[18F]fluoro-D-glucose ([^18^F]FDG) in assessing tumor response, guiding radiation treatment planning, and in treatment decision-making [8,9,10,11,12]. One such study, which we performed and can be considered a predecessor to this study, provided valuable clinical insights. It revealed that certain pretreatment [^18^F]FDG PET/CT parameters were significantly associated with clinical outcomes in patients with recurrent NSCLC treated with high-dose reirradiation [13].

Studies have demonstrated that changes in [^18^F]FDG PET/CT parameters before, during, and after initial curative chemoradiotherapy correlate with clinical outcomes [8,14,15,16]. However, it is important to point out that the correlation between delta changes in [^18^F]FDG PET/CT parameters before the first and second radiation treatments, as well as their subsequent impact on clinical outcomes in recurrent lung cancer patients, has not been investigated to date. Understanding these correlations may guide the development of more personalized treatment protocols and improve selection for recurrent lung cancer patients undergoing thoracic reirradiation.

Therefore, the aim of our retrospective study was to investigate the significance of delta changes in [^18^F]FDG PET/CT parameters for clinical outcomes (LCR, PFS and OS) in NSCLC patients who underwent high-dose thoracic reirradiation.

## 2. Methods

### 2.1. Patients

We included 47 patients who underwent high-dose reirradiation at our institute between 2013 and 2021. Patients were staged according to the 8th TNM edition and had to meet the following criteria: (1) Primary and secondary tumors were located in the lungs and all patients received two radiation treatments with curative intent. (2) All patients were evaluated by our multidisciplinary tumor board and were deemed inoperable. (3) The primary tumor was histologically verified as NSCLC. (4) [^18^F]FDG PET/CT was mandatory for the diagnosis of initial as well as recurrent tumors. (5) ECOG was less than or equal to 2. (6) The interval between radiation treatments was at least nine months. We made an exception for one patient who was reirradiated 6 months after the first irradiation for lack of other treatment options (neither systematic nor surgical) and the time interval was not less than the recommended minimum [4]. Patients who had undergone palliative or postoperative radiotherapy, or those with out-of-field recurrences were excluded from the analysis.

### 2.2. Therapy

For treatment planning we used a planning CT with a rotation time of 1 s and a pitch of 0.35 for the IMRT/VMAT technique, or four-dimensional computed tomography (4D-CT) for stereotactic ablative body radiotherapy (SABR). All patients were immobilized with a vacuum cradle and WingSTEP for the arms. The planning CT was registered to the [^18^F]FDG PET-CT in the TPS using a deformable registration. We administered IMRT/VMAT either conventionally with 2 Gy per fraction, in a hypofractionated regimen (one fraction of 3 Gy per day), or dose-differentiated accelerated in twice-daily fractions of 1.8 Gy (DART-bid).

The SABR treatment was administered in two distinct regimens. The first regimen was delivered in eight fractions of 8 Gy (prescribed to the surrounding 65% isodose) on a daily basis to central tumors, defined as those located within 2 cm of the proximal bronchial tree. The second regimen, which was used for peripheral tumors, was delivered in three fractions of 15.4 Gy (prescribed to the surrounding 65% isodose) every other day.

For systemic therapy, two cycles of either cisplatin (75 mg/m^2^) in combination with pemetrexed (500 mg/m^2^) or gemcitabine (1000 mg/m^2^) were administered prior to reirradiation, based on tumor histology. Following the second course of radiation, one of the four most common immunotherapeutics for thoracic cancers, i.e., durvalumab, atezolizumab, pemrolizumab, or nivolumab. was administered.

### 2.3. Organs at Risk (OAR) and Toxicity

Cumulative dose was calculated using dose–volume histograms at first and second radiation for each organ at risk. Cumulative dose constraints are described elsewhere [17]. Since various fractionation regimens were used, total radiation doses were compared by biologically equivalent dose in 2 Gy fractions (EQD2). An alpha/beta value of 10 was selected for the tumor.

$${EQD}_{2}=D\times \frac{d+\frac{\alpha }{\beta }}{2+\frac{\alpha }{\beta }}$$

Common Terminology Criteria for Adverse Events (CTCAE) version 5.0 was used to assess toxicity. As grade 1 is defined as toxicity for which no medication is required, toxicity was evaluated from grade 2 onwards.

### 2.4. Follow-Up

Initial follow-up was at six weeks post radiation, then in 3-month intervals up to 2 years and 6-month intervals for the following 3 years. Contrast-enhanced computed tomography (CT) scans and pulmonary function tests were performed at each follow-up. Tumor recurrence was assessed according to the RECIST 1.1 criteria. Patients in whom local recurrence or new lesions were suspected on thoracic CT underwent [^18^F]FDG PET/CT.

### 2.5. Delta [^18^F]FDG PET/CT Metrics Calculation

We expressed the delta values in percentages ($relative\;Delta\;(percent)=\frac{baseline\;metric\;at\;second\;radiation-baseline\;metric\;at\;first\;radiation}{baseline\;metric\;at\;first\;radiation}\times 100$), analogous to the published literature [18,19,20,21,22], and we additionally calculated them in absolute numbers as well (absolute Delta = baseline metric at second radiation minus baseline metric at first radiation).

### 2.6. [^18^F]FDG PET/CT

The [^18^F]FDG PET/CT imaging procedure was carried out using integrated PET/CT systems, following established EANM/SNMMI guidelines for tumor imaging, spanning from the head to the feet (extended whole body examination). Participants were required to fast for a period of 4 to 6 h prior to receiving an intravenous dose of [^18^F]FDG at a concentration of 4 MBq/kg (0.14 mCi/kg), with blood glucose levels verified to be under 150 mg/dL. After an average uptake period of 60 ± 5 min post-injection, a low-dose, non-contrast CT scan was performed to aid in anatomical alignment and to correct PET images for attenuation.

Quantitative analysis and lesion delineation were carried out by a board-certified nuclear medicine physician. Metabolic tumor volume (MTV) was assessed using a semi-automated delineation method based on a percentage threshold derived from the spherical volume of interest (VOI). To accurately define the lesion boundaries and avoid including nearby healthy tissues, a threshold range of 40–60% was selected for optimal contouring. To ensure accuracy, all semi-automatically generated VOIs were visually inspected and adjusted as necessary. Adjacent physiologic activities were carefully excluded from the defined regions. Key metabolic parameters from [^18^F]FDG PET/CT, such as MTV, total lesion glycolysis (TLG), and the most clinically relevant standardized uptake values (SUVs)—SUVmax, SUVpeak, SUVmean, SULpeak, and SULmax—were measured for all detected lesions. In this analysis, [^18^F]FDG-PET metrics for the primary tumor and lymph nodes were evaluated separately, in alignment with our prior study [13].

### 2.7. Statistical Analysis

We used multivariate analysis (MVA) with a forward stepwise Cox regression to determine the correlation between the change (delta) in quantitative PET metrics of interest and LRC, PFS, and OS for primary tumor and lymph nodes separately. To avoid co-linearity between PET metrics, we analyzed all parameters (MTV, TLG, SUVmax and SULpeak) in a separate manner. As covariates we included the patient characteristics (see Table 1) and treatment-related factors (see Table 2). For patient stratification, we used a cutoff defined as any increase (difference above zero, delta > 0) in [^18^F]FDG PET/CT metrics between first irradiation and reirradiation and compared them in relation to LRC, PFS, and OS using Cox regression and the log-rank test with the Kaplan–Meier estimator. LRC, PFS, and OS were defined as follows: LRC was the time from the end of reirradiation to local or regional recurrence, with local recurrence indicated by tumor growth within the reirradiated volume (for IMRT/VMAT at least within the 80% Isodose, and for SABR within the 65% Isodose). Regional recurrence was defined as tumor recurrence in the regional lymphatic drainage system. PFS was measured from the end of reirradiation until local or regional recurrence and/or distant metastasis or death. OS was calculated from the end of treatment to death or until last follow-up.

## 3. Results

The study included 47 patients (Table 1). Of these, 34 (72%) were male and 13 (28%) female, with a median age of 67 years (range: 57–83). All patients had histologically confirmed NSCLC at initial diagnosis. Squamous cell carcinoma was diagnosed in 24 (51%) patients, adenocarcinoma in 19 (40%) patients, and not otherwise specified (NOS) in 4 (9%) patients. As shown in Table 2, slightly more than half of the tumor recurrences occurred centrally (24 patients; 51%); the other half were peripheral (23 patients; 49%). Regarding UICC stage, more than half (25 patients; 53%) were classified as stage III. Stage I and II together comprised 18 patients (38%). A total of 4 patients (9%) were classified as UICC stage IV (oligometastatic), all of whom received ablative reirradiation. A total of 28 patients (60%) received retreatment with radioimmunotherapy (with or without chemotherapy), 12 patients (25%) received retreatment with radiation alone, and 7 patients (15%) received retreatment with radiochemotherapy. The median planning target volume (PTV) at reirradiation was 50 mL (range: 4.5–239 mL). The median cumulative EQD2s delivered to the tumor and lymph nodes were 133 Gy (range: 108–249 Gy) and 105 Gy (86–144 Gy), respectively, with a median time between first and reirradiation of 18 months (range: 6–80 months). The majority of patients tolerated the reirradiation very well. Acute radiation toxicities, grade 2 and 3, were observed in 6/47 (13%) and 2/47 (4%) patients, respectively. The most relevant single toxicities were esophagitis (*n* = 4, 8.5%) and pneumonitis (*n* = 4, 8.5%) The sole observed grade 2+ late adverse effect was hemorrhage, which occurred in 2 patients (4%). No acute or late grade 4/5 toxicities were found (Table 3). The cumulative radiation doses to the organs at risk are summarized in Table 4.

**Figure 1:**
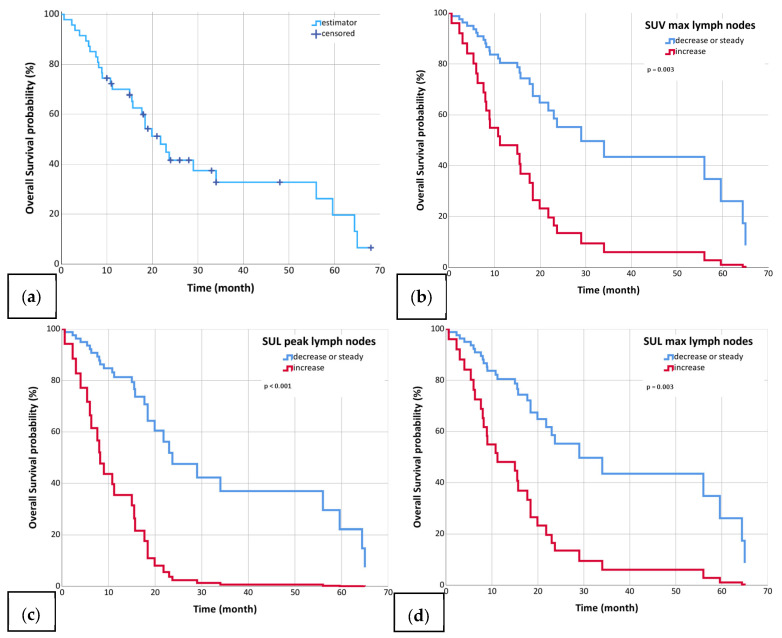
Diagram (**a**) shows overall survival (median 21.8 months) in the whole cohort (*n* = 47, (**a**)). Diagrams (**b**–**d**) show overall survival for patients with increased (blue) and those with decreased or unchanged (red) SUV max in lymph nodes (*n* = 47, (**b**)), SULpeak in lymph nodes (*n* = 44, (**c**)), and SULmax in lymph nodes (*n* = 47, (**d**)) at first radiation and reirradiation.

**Figure 2:**
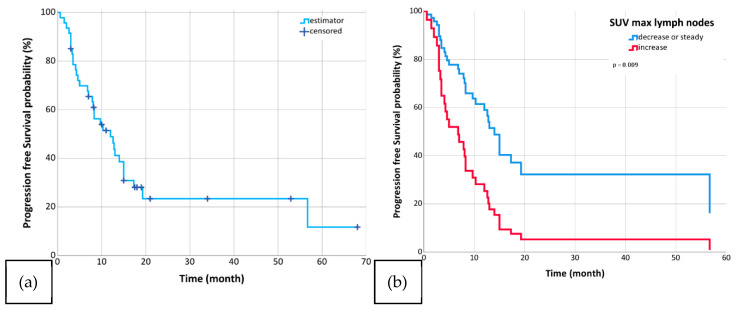

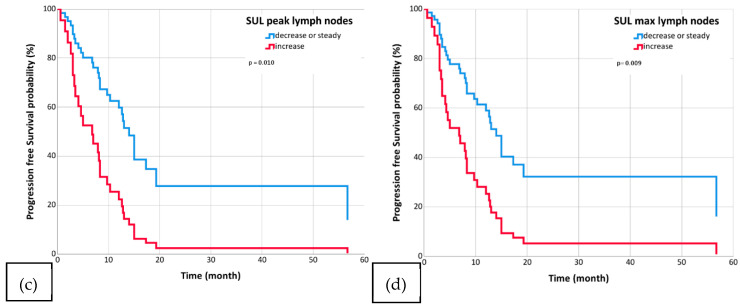
Diagram (**a**) shows Progression-Free Survival (median 12 months) in the whole cohort (*n* = 47, (**a**)). Diagrams (**b**–**d**) show Progression-Free Survival for patients with increased (blue) and those with decreased or unchanged (red) SUV max in lymph nodes (*n* = 47, (**b**)), SULpeak in lymph nodes (*n* = 44, (**c**)), and SULmax in lymph nodes (*n* = 47, (**d**)) at first radiation and reirradiation.

**Figure 3:**
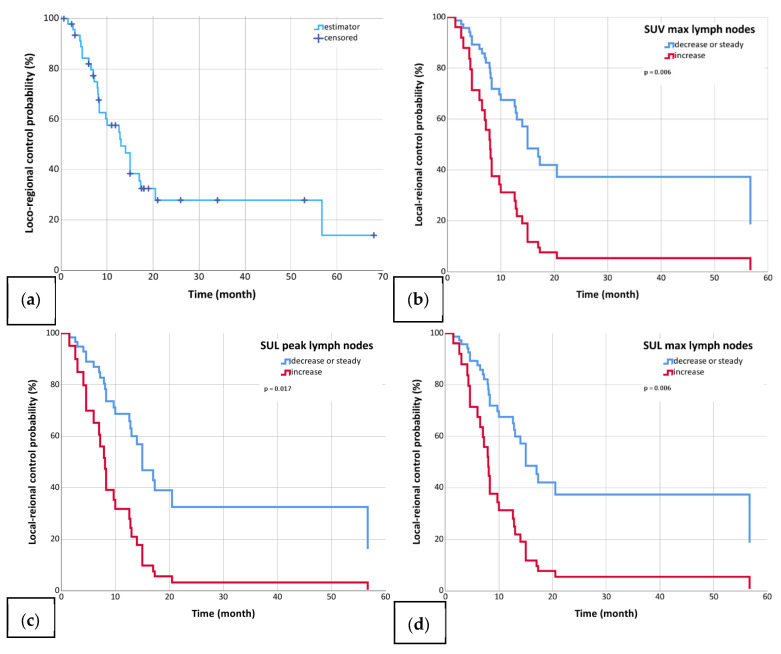
Diagram (**a**) shows locoregional control (median 13 months) in the whole cohort (*n* = 47, (**a**)). Diagrams (**b**–**d**) show locoregional control for patients with increased (blue) and those with decreased or unchanged (red) SUV max in lymph nodes (*n* = 47, (**b**)), SULpeak in lymph nodes (*n* = 44, (**c**)), and SULmax in lymph nodes (*n* = 47, (**d**)) at first radiation and reirradiation.

Patients were followed for a median time of 33 months (range: 0.6–68 months) after reirradiation. The mOS was 21.8 months (95%-CI: 16.3–27.3; Figure 1), the mPFS was 12 months (95%-CI: 6.7–17.3; Figure 2), and the mLRC was 13 months (95%-CI: 9.0–17.0; Figure 3). MVA revealed that the delta changes in the SULpeak, SUVmax, and SULmax of the metastatic lymph nodes significantly impacted OS (SULpeak *p* < 0.001, HR = 5.0; 95%-CI = 2.0–12.6; SUV max *p* = 0.003, HR = 3.4; 95%-CI = 1.5–7.6; SULmax *p* = 0.003, HR = 3.4; 95%-CI = 1.5–7.6; Table 5), PFS (SULpeak *p* = 0.010, HR = 2.9; 95%-CI = 1.3–6.6; SUVmax *p* = 0.009, HR = 2.6; 95%-CI = 1.3–5.3; SULmax *p* = 0.009, HR = 2.6; 95%-CI = 1.3–5.3; Table 6), and LRC (SULpeak *p* = 0.017, HR = 3.1; 95%-CI = 1.2–7.6; SUVmax *p* = 0.006, HR = 3.0; 95%-CI = 1.4–6.4; SULmax *p* = 0.006, HR = 3.0; 95%-CI = 1.4–6.4; Table 7). In addition, delta changes in MTV and TLG of the lymph nodes significantly impacted OS (MTV *p* = 0.028, HR = 2.6; 95%-CI = 1.1–6.0; TLG *p* = 0.028, HR = 2.6; 95%-CI = 1.1–6.0) but were not significant for either LRC or PFS (Table 5, Table 6 and Table 7). The only non-PET/CT parameter that significantly correlated with clinical outcomes was histology (see Table 5, Table 6 and Table 7).

## 4. Discussion

Our study found that delta changes in metastatic lymph node SUVmax, SULmax, and SULpeak significantly impacted all measured clinical endpoints (LRC, PFS, and OS). The results indicate that these parameters can potentially provide valuable prognostic information and could assist in optimizing treatment choices. It should be mentioned, however, that this is the first study of its kind, and as such, comparisons were made with publication data obtained from primary treatment settings. In line with these analyses, we investigated the recurrent primary tumor and lymph nodes separately, analogous to previously published studies [13,19]. The reason for this was to account for intertumoral heterogeneity, defined as heterogeneity between two distinct lesions [23], which is known to be positively correlated with tumor aggressiveness [24]. Indeed, our results showed that delta [^18^F]FDG-PET parameters of the metastatic lymph nodes, but not those of the primary tumor recurrence, impacted clinical outcomes. Mena et al. [23] identified several factors that affect tissue microenvironment and can lead to tumor heterogeneity, two of which were angiogenesis and hypoxia. In this context, Ji et al. [25] described the microenvironment specifically of the metastatic lymph nodes, which undergo remodeling due to tumor infiltration. This includes abnormal blood flow and inadequate lymphatic valve function, resulting in increased interstitial pressure that can induce hypoxia [25], a known limiting factor for radiation efficacy [23]. Altered oxygenation as a radiobiological cornerstone could—at least partially—contribute to an explanation for our clinical results. Furthermore, lymph nodes are anatomically a key gateway of tumor cell migration leading to metastasis, and have already been demonstrated to be prognostic factors for disease progression affecting all clinical endpoints [26]. Additionally, the enhanced immune response to tumor cells, which initially occurs in the lymph nodes as the center of immune regulation [25], resulting in high metabolic activity and increased [^18^F]FDG uptake [11], could potentially make them a more suitable prognostic surrogate than the recurrent primary tumor itself. We measured the [^18^F]FDG-PET parameters at two distinct time points, namely before first radiation and before reirradiation, and our cutoff point was any difference above zero (>0) between them. In our opinion, this is clinically applicable considering that most centers do not have sufficient resources to perform close surveillance after first radiation with [^18^F]FDG-PET and mostly use it only when there is suspicion of disease recurrence or progression. An increasing pattern in delta SUVmax, SULmax, and SULpeak reflects the higher metabolic activity in recurrent tumor tissue, which could be an indication of alterations in the tumor microenvironment. This might be driven by the selection of more resistant tumor cells after the primary treatment, which could contribute to a more aggressive phenotype in recurrent tumors [6]. Such higher aggressiveness often translates into increased therapy resistance, which could be the reason for poorer locoregional control, shorter PFS, and poorer OS in patients with greater delta changes in [^18^F]FDG-PET metrics, as demonstrated in our study. Several studies have examined the prognostic value of delta changes in PET/CT parameters, particularly SUVmax, in patients with stage III NSCLC, but in the primary setting.

In contrast to our study, these studies describe a short-term metabolic response after radiotherapy, whereas our analysis of the baseline-to-recurrence changes in [^18^F]FDG-PET-metrics investigate the long-term metabolic changes in tumor cells that survived the initial radiation treatment. Although the timing of assessment differs, both approaches are based on the same biological principle: changes in [^18^F]FDG-PET metrics reflect clinically meaningful alterations in tumor metabolism, which is an important component of tumor biology and therefore, as mentioned above, could predict tumor behavior. In this context, a study of 49 patients with stage III NSCLC who underwent primary chemoradiation found that the delta changes in SUVmax for both primary tumor and metastatic lymph nodes were significantly associated with OS (HR, 0.06; *p* = 0.002), DFS (HR, 0.03; *p* = 0.001), locoregional control (HR, 0.04; *p* = 0.002), and distant metastasis-free survival (HR, 0.07; *p* = 0.028) [16]. Similarly, research by Tang and Colleagues in a cohort of locally advanced NSCLC patients treated with chemotherapy, radiotherapy, and immunotherapy also demonstrated a significant association between delta SUVmax and OS [18]. Another study, focused on stage III NSCLC patients with primary radiochemotherapy, found that delta SUVmax was significantly associated with both locoregional and distant progression [20]. A similar study showed that delta SUVmax before and after initial radiation treatment significantly impacted OS, local, and regional failure, although this was more pronounced for the primary tumor than the lymph nodes, in contrast to our findings [19]. Data on delta SULmax is scarce, even in the primary setting. However, given the strong collinearity between SULmax and SUVmax as described in the literature [27] and shown by our results, and the already established superiority of SULmax over SUVmax [9], it is reasonable to conclude that findings related to delta SUVmax could also be applicable to delta SULmax. However, SULpeak, which represents the highest uptake voxel within a standardized spherical region and has already been established as a treatment response parameter in the PERCIST (PET Response Criteria in Solid Tumors) criteria [9], could serve as an even more suitable imaging biomarker. It provides a more comprehensive metabolic profile, particularly in recurrent lung cancer tumors [13], which are characterized by greater intra- and intertumoral heterogeneity, leading to increased aggressiveness [6,13]. This may explain the strong association between delta SULpeak and clinical outcomes in our study. Additionally, a study investigating the prognostic value of [^18^F]FDG-PET parameters in locally advanced NSCLC identified delta SULpeak as a significant prognosticator of clinical outcomes in the primary setting, further supporting our findings [28].

All of these comparisons should be taken with caution, particularly because they are based on data from the primary setting, which is a crucial difference compared to our study. Furthermore, with the exception of two studies [16,19], all of them investigated the [^18^F]FDG-PET metrics for both the primary tumor and the lymph nodes together. Another major limitation of the current analysis is both its retrospective nature as well as the heterogeneity of the patient population, which reflects daily clinical routine.

Nevertheless, compared to other studies, our cohort size is higher than in most published literature on recurrent lung cancer patients who underwent reirradiation [2,3,5,29]. In addition, these delta [^18^F]FDG-PET metrics may facilitate a more selective identification of patients with poor outcomes who could benefit from tailored treatment approaches or alternative therapies.

## 5. Conclusions

Delta changes in SULpeak, SUVmax, and SULmax of the metastatic lymph nodes significantly affected all clinical endpoints (OS, PFS, and LRC) in recurrent NSCLC patients treated with reirradiation–chemoimmunotherapy. While these findings support the use of [^18^F]FDG-PET-based metrics for prognostication, which could help guide individualized treatment strategies, further prospective studies with larger cohorts are needed for validation.

## Figures and Tables

**Table 1:** Patient characteristics at reirradiation.

		Patients N = 47	
Characteristics at reirradiation	Age (years)	median	67
	Sex	male	34
		female	13
	Weight loss (%)	>5%	17
		<5%	30
	Histology	adeno	19
		squamous	24
		NOS	4
	UICC-stage	I	12
		II	6
		III	25
		IV	4
	Charlson Comorbidity Index range: 2–9	median	5

**Table 2:** Treatment related factors at reirradiation.

Patients N = 47			
Treatment-related factors	PTV of recurrent tumor and lymph nodes in mL range: 4.5–239	median	50
	Tumor location	peripheral	23
		central	24
	Treatment concept (n)	CRTX	7
		(C)RIT	28
		RT alone	12
	Cumulative EQD2 tumor (Gy) range: 108–249	median	133
	Cumulative EQD2 lymph nodes (Gy) range: 86–144	median	105

**Table 3:** Treatment-related toxicity.

Toxicity (N = 47)						
Type of Toxicity		Grade 1	Grade 2	Grade 3	Grade 4	Grade 5
Acute	Esophagitis	n.a.	3	1	0	0
	Pneumonitis	n.a.	3	1	0	0
Late	Esophagitis	n.a.	0	0	0	0
	Heart tox.	n.a.	0	0	0	0
	Chest wall pain	n.a.	0	0	0	0
	Hemorrhage	n.a.	1	1	0	0

**Table 4:** Dosimetric cumulative data of organs at risk.

Patients N = 47			
Dosimetric cumulative data	MLD (Gy) range: 3.4–24.3	median	15.4
	V20 total lung (%) range: 3–50	median	26.5
	Mean esophageal dose (Gy) range: 2–48	median	23.3
	Aorta Dmax (Gy) range: 12–128	median	80
	Pulmonary trunk and pulmonary arteries Dmax (Gy) range: 9–130	median	89
	Spinal cord Dmax (Gy) range: 8–68.6	median	42
	Heart V25 (Gy) range: 0.5–75	median	8.5

**Table 5:** Uni- and multivariate analyses (corresponding *p*-values shown below) of recurrent primary tumor’s (referred to as primary in the table) and lymph nodes’ (LN) volumetric and intensity 18F-FDG-PET metrics with patient characteristics, histology, and treatment-related factors at reirradiation (Cox regression) related to overall survival (OS). The abbreviations used in this table are as follows: n.a. = Not Assessed, n.s. = Not Significant.

Overall Survival (OS)																				
Variables	Primary (*n* = 47)	LN (*n* = 46)			Primary (*n* = 47)		LN (*n* = 46)		Primary (*n* = 44)		LN (*n* = 44)		Primary (*n* = 47)		LN (*n* = 47)		Primary (*n* = 47)		LN (*n* = 47)	
	UVA	MVA	UVA	MVA	UVA	MVA	UVA	MVA	UVA	MVA	UVA	MVA	UMA	MVA	UVA	MVA	UVA	MVA	UVA	MVA
Tumor location	0.201	n.s.	0.131	n.s.	0.201	n.s.	0.131	n.s.	0.264	n.s.	0.263	n.s.	0.201	n.s.	0.201	n.s.	0.201	n.s.	0.201	n.s.
Age	0.953	n.s.	0.928	n.s.	0.953	n.s.	0.928	n.s.	0.787	n.s.	0.883	n.s.	0.953	n.s.	0.953	n.s.	0.953	n.s.	0.953	n.s.
Sex	0.569	n.s.	0.494	n.s.	0.569	n.s.	0.494	n.s.	0.818	n.s.	0.434	n.s.	0.569	n.s.	0.569	n.s.	0.569	n.s.	0.569	n.s.
Weight loss	0.552	n.s.	0.654	n.s.	0.552	n.s.	0.654	n.s.	0.643	n.s.	0.971	n.s.	0.552	n.s.	0.552	n.s.	0.552	n.s.	0.552	n.s.
Histology	0.045	n.s.	0.069	n.s.	0.045	n.s.	0.069	n.s.	0.069	n.s.	0.077	n.s.	0.045	n.s.	0.045	n.s.	0.045	n.s.	0.045	n.s.
UICC	0.070	n.s.	0.061	n.s.	0.070	n.s.	0.061	n.s.	0.139	n.s.	0.054	n.s.	0.070	n.s.	0.070	n.s.	0.070	n.s.	0.070	n.s.
Charlson Comorbidity Index	0.220	n.s.	0.301	n.s.	0.220	n.s.	0.301	n.s.	0.295	n.s.	0.469	n.s.	0.220	n.s.	0.220	n.s.	0.220	n.s.	0.220	n.s.
PTV (ml)	0.435	n.s.	0.401	n.s.	0.435	n.s.	0.401	n.s.	0.405	n.s.	0.434	n.s.	0.435	n.s.	0.435	n.s.	0.435	n.s.	0.435	n.s.
Systemic therapy	0.696	n.s.	0.774	n.s.	0.696	n.s.	0.774	n.s.	0.509	n.s.	0.767	n.s.	0.696	n.s.	0.696	n.s.	0.696	n.s.	0.696	n.s.
EQD2 tumor total	0.128	n.s.	0.109	n.s.	0.128	n.s.	0.109	n.s.	0.195	n.s.	0.097	n.s.	0.128	n.s.	0.128	n.s.	0.128	n.s.	0.128	n.s.
EQD2 LN total	0.155	n.s.	0.099	n.s.	0.155	n.s.	0.099	n.s.	0.305	n.s.	0.088	n.s.	0.155	n.s.	0.155	n.s.	0.155	n.s.	0.155	n.s.
MTV	0.816	n.s.	0.023	0.028	n.a.	n.a.	n.a.	n.a.	n.a.	n.a.	n.a.	n.a.	n.a.	n.a.	n.a.	n.a.	n.a.	n.a.	n.a.	n.a.
TLG	n.a.	n.a.	n.a.	n.a.	0.576	n.s.	0.023	0.028	n.a.	n.a.	n.a.	n.a.	n.a.	n.a.	n.a.	n.a.	n.a.	n.a.	n.a.	n.a.
SULpeak	n.a.	n.a.	n.a.	n.a.	n.a.	n.a.	n.a.	n.a.	0.165	n.s.	<0.001	<0.001	n.a.	n.a.	n.a.	n.a.	n.a.	n.a.	n.a.	n.a.
SUV max	n.a.	n.a.	n.a.	n.a.	n.a.	n.a.	n.a.	n.a.	n.a.	n.a.	n.a.	n.a.	0.079	n.s.	0.002	0.003	n.a.	n.a.	n.a.	n.a.
SULmax	n.a.	n.a.	n.a.	n.a.	n.a.	n.a.	n.a.	n.a.	n.a.	n.a.	n.a.	n.a.	n.a.	n.a.	n.a.	n.a.	0.097	n.s.	0.002	0.003

**Table 6:** Uni- and multivariate analyses (corresponding *p*-values shown below) of recurrent primary tumor’s (referred to as primary in the table) and lymph nodes’ (LN) volumetric and intensity 18F-FDG-PET metrics with patient characteristics, histology, and treatment-related factors at reirradiation (Cox regression) related to progression-free survival (PFS). The abbreviations used in this table are as follows: n.a. = Not Assessed, n.s. = Not Significant.

Progression Free Survival (PFS)																				
Variables	Primary (*n* = 47)		LN (*n* = 46)		Primary (*n* = 47)		LN (*n* = 46)		Primary (*n* = 44)		LN (*n* = 44)		Primary (*n* = 47)		LN (*n* = 47)		**Primary (*n* = 47)**		**LN (*n* = 47)**	
	UVA	MVA	UVA	MVA	UVA	MVA	UVA	MVA	UVA	MVA	UVA	MVA	UMA	MVA	UVA	MVA	**UVA**	**MVA**	**UVA**	**MVA**
Tumor location	0.276	n.s.	0.340	n.s.	0.276	n.s.	0.340	n.s	0.244	0.016	0.425	n.s.	0.276	n.s.	0.276	n.s.	0.276	n.s.	0.276	n.s.
Age	0.204	n.s.	0.211	n.s.	0.204	n.s.	0.211	n.s	0.249	n.s.	0.212	n.s.	0.204	n.s.	0.204	n.s.	0.204	n.s.	0.204	n.s.
Sex	0.317	n.s.	0.356	n.s.	0.317	n.s.	0.356	n.s	0.248	n.s.	0.356	n.s.	0.317	n.s.	0.317	n.s.	0.317	n.s.	0.317	n.s.
Weight loss	0.561	n.s.	0.480	n.s.	0.561	n.s.	0.480	n.s	0.664	n.s.	0.642	n.s.	0.561	n.s.	0.561	n.s.	0.561	n.s.	0.561	n.s.
Histology	0.024	0.031	0.012	0.018	0.024	0.031	0.012	0.018	0.022	0.029	0.038	n.s.	0.024	0.031	0.024	n.s.	0.024	0.031	0.024	n.s.
UICC	0.094	n.s.	0.099	n.s.	0.094	n.s.	0.099	n.s	0.112	n.s.	0.058	n.s.	0.094	n.s.	0.094	n.s.	0.094	n.s.	0.094	n.s.
Charlson Comorbidity Index	0.235	n.s.	0.147	n.s.	0.235	n.s.	0.147	n.s	0.340	n.s.	0.305	n.s.	0.235	n.s.	0.235	n.s.	0.235	n.s.	0.235	n.s.
PTV (ml)	0.601	n.s.	0.609	n.s.	0.601	n.s.	0.609	n.s	0.500	n.s.	0.721	n.s.	0.601	n.s.	0.601	n.s.	0.601	n.s.	0.601	n.s.
Systemic therapy	0.928	n.s.	0.958	n.s.	0.928	n.s.	0.958	n.s	0.957	n.s.	0.683	n.s.	0.928	n.s.	0.928	n.s.	0.928	n.s.	0.928	n.s.
EQD2 tumor total	0.624	n.s.	0.648	n.s.	0.624	n.s.	0.648	n.s	0.837	n.s.	0.697	n.s.	0.624	n.s.	0.624	n.s.	0.624	n.s.	0.624	n.s.
EQD2 LN total	0.111	n.s.	0.143	n.s.	0.111	n.s.	0.143	n.s	0.131	n.s.	0.118	n.s.	0.111	n.s.	0.111	n.s.	0.111	n.s.	0.111	n.s.
MTV	0.652	n.s.	0.027	n.s.	n.a	n.a	n.a.	n.a.	n.a.	n.a.	n.a.	n.s.	n.a.	n.a.	n.a.	n.a.	n.a.	n.a.	n.a.	n.a.
TLG	n.a.	n.a.	n.a.	n.a.	0.945	n.s.	0.027	n.s	n.a.	n.a.	n.a.	n.a.	n.a.	n.a.	n.a.	n.a.	n.a.	n.a.	n.a.	n.a.
SULpeak	n.a.	n.a.	n.a.	n.a.	n.a	n.a.	n.a.	n.a.	0.032	n.s.	0.007	0.010	n.a.	n.a.	n.a.	n.a.	n.a.	n.a.	n.a.	n.a.
SUV max	n.a.	n.a.	n.a.	n.a.	n.a	n.a.	n.a.	n.a.	n.a.	n.a.	n.a.	n.a.	0.053	n.s.	0.007	0.009	n.a.	n.a.	n.a.	n.a.
SULmax	n.a.	n.a.	n.a.	n.a.	n.a.	n.a.	n.a.	n.a.	n.a.	n.a.	n.a.	n.a.	n.a.	n.a.	n.a.	n.a.	0.072	n.s.	0.007	0.009

**Table 7:** Uni- and multivariate analyses (corresponding *p*-values shown below) of recurrent primary tumor’s (referred to as primary in the table) and lymph nodes’ (LN) volumetric and intensity 18F-FDG-PET metrics with patient characteristics, histology, and treatment-related factors at reirradiation (Cox regression) related to locoregional control (LRC). The abbreviations used in this table are as follows: n.a. = Not Assessed, n.s. = Not Significant.

Locoregional Control (LRC)																				
Variables	Primary (*n* = 47)		LN (*n* = 46)		Primary (*n* = 47)		LN (*n* = 46)		Primary (*n* = 44)		LN (*n* = 44)		Primary (*n* = 47)		**LN (*n* = 47)**		**Primary (*n* = 47)**		**LN (*n* = 47)**	
	UVA	MVA	UVA	MVA	UVA	MVA	UVA	MVA	UVA	MVA	UVA	MVA	UVA	MVA	**UVA**	**MVA**	**UVA**	**MVA**	**UVA**	**MVA**
Tumor location	0.524	n.s.	0.604	n.s.	0.524	n.s.	0.604	n.s.	0.434	n.s.	0.747	n.s.	0.524	n.s.	0.524	n.s.	0.524	n.s.	0.524	n.s.
Age	0.126	n.s.	0.125	n.s.	0.126	n.s.	0.125	n.s.	0.157	n.s.	0.125	n.s.	0.126	n.s.	0.126	n.s.	0.126	n.s.	0.126	n.s.
Sex	0.567	n.s.	0.627	n.s.	0.567	n.s.	0.627	n.s.	0.429	n.s.	0.664	n.s.	0.567	n.s.	0.567	n.s.	0.567	n.s.	0.567	n.s.
Weight loss	0.386	n.s.	0.331	n.s.	0.386	n.s.	0.331	n.s.	0.479	n.s.	0.478	n.s.	0.386	n.s.	0.386	n.s.	0.386	n.s.	0.386	n.s.
Histology	0.055	n.s.	0.035	0.046	0.055	n.s.	0.035	0.046	0.037	0.049	0.104	n.s.	0.055	n.s.	0.055	n.s.	0.055	n.s.	0.055	n.s.
UICC	0.065	n.s.	0.069	n.s.	0.065	n.s.	0.069	n.s.	0.076	n.s.	0.040	n.s.	0.065	n.s.	0.065	n.s.	0.065	n.s.	0.065	n.s.
Charlson Comorbidity Index	0.827	n.s.	0.754	n.s.	0.827	n.s.	0.754	n.s.	0.874	n.s.	0.928	n.s.	0.827	n.s.	0.827	n.s.	0.824	n.s.	0.824	n.s.
PTV (ml)	0.426	n.s.	0.427	n.s.	0.426	n.s.	0.427	n.s.	0.336	n.s.	0.520	n.s.	0.426	n.s.	0.426	n.s.	0.426	n.s.	0.426	n.s.
Systemic therapy	0.732	n.s.	0.775	n.s.	0.732	n.s.	0.775	n.s.	0.750	n.s.	0.366	n.s.	0.732	n.s.	0.732	n.s.	0.732	n.s.	0.732	n.s.
EQD2 tumor total	0.798	n.s.	0.824	n.s.	0.798	n.s.	0.824	n.s.	0.983	n.s.	0.874	n.s.	0.798	n.s.	0.798	n.s.	0.798	n.s.	0.798	n.s.
EQD2 LN total	0.092	n.s.	0.114	n.s.	0.092	n.s.	0.114	n.s.	0.103	n.s.	0.095	n.s.	0.092	n.s.	0.092	n.s.	0.092	n.s.	0.092	n.s.
MTV	0.433	n.s.	0.038	n.s.	n.a.	n.a.	n.a.	n.a.	n.a.	n.a.	n.a.	n.a.	n.a.	n.a.	n.a.	n.a.	n.a.	n.a.	n.a.	n.a.
TLG	n.a.	n.a	n.a.	n.a.	0.656	n.s.	0.038	n.s.	n.a.	n.a.	n.a.	n.a.	n.a.	n.a.	n.a.	n.a.	n.a.	n.a.	n.a.	n.a.
SULpeak	n.a.	n.a	n.a.	n.a.	n.a.	n.a	n.a.	n.a.	0.276	n.s.	0.012	0.017	n.a.	n.a.	n.a.	n.a.	n.a.	n.a.	n.a.	n.a.
SUV max	n.a.	n.a	n.a.	n.a.	n.a.	n.a	n.a.	n.a.	n.a.	n.a.	n.a.	n.a.	0.151	n.s.	0.004	0.006	n.a.	n.a.	n.a.	n.a.
SULmax	n.a.	n.a	n.a.	n.a	n.a.	n.a	n.a.	n.a	n.a.	n.a	n.a.	n.a	n.a.	n.a	n.a.	n.a	0.229	n.s.	0.004	0.006

## Data Availability

The datasets used and/or analyzed during the current study are available from the corresponding author on reasonable request. Access to the data may be restricted due to privacy, legal, and ethical considerations.

## Abbreviations

CCI	Charlson comorbidity index
CTCAE	Common toxicity criteria for adverse events
DART	Dose-differentiated accelerated radiotherapy
ECOG	Eastern cooperative oncology group
EQD2	Biologically equivalent dose in 2 Gy fractions
FDG-PET-CT	Fluorodeoxyglucose-positron emission tomography
IMRT	Intensity modulated radiotherapy
LRC	Locoregional control
TLG	Total lesion glycolysis
mOS	Median overall survival
MTV	Metabolic tumor volume
MVA	Multivariate analysis
NSCLC	Non-small cell lung cancer
OAR	Organs at risk
OS	Overall survival
PFS	Progression free survival
PS	Performance score
RT	Radiotherapy
SABR	Stereotactic ablative body therapy
SCLC	Small-cell lung cancer
SULmax	Lean body mass corrected SUV max
SULpeak	Lean body mass corrected
SUV peak	Standardized uptake value peak
SUVmax	Maximum standardized uptake value
SUVmean	Mean standardized uptake value
SUVpeak	Peak standardized uptake value
TPS	Treatment planning system
